# Editorial: DNA methylation: The aging clock

**DOI:** 10.3389/fcell.2023.1164429

**Published:** 2023-03-15

**Authors:** Barbara Ghinassi, Maria R. Matarazzo, Annalisa Di Ruscio

**Affiliations:** ^1^ Anatomy and Cell Differentiation Lab, Department of Medicine and Aging Sciences, “G. D’Annunzio”University of Chieti—Pescara, Chieti, Italy; ^2^ Institute of Genetics and Biophysics Adriano Buzzati Traverso, (IGB-ABT) CNR, Naples, Italy; ^3^ Harvard Medical School Initiative for RNA Medicine, Harvard Medical School, Boston, MA, United States; ^4^ Department of Medicine and Cancer Research Institute, Beth Israel Deaconess Medical Center, Harvard Medical School, Boston, MA, United States

**Keywords:** DNA methylation, epigenetic clock, aging, alzheimer disease, metabolic disorders, breast cancer

One of the most fascinating biological questions is “why do we age?” Chronological age is not a reliable measure to assess the aging process. To understand and control aging, we need tools and approaches to evaluate biological age. A plethora of studies in both human and murine models have shown that aging is associated with changes in DNA methylation, a key epigenetic signature implicated in the regulation of gene expression. More recently, the definition of the “epigenetic clocks” - a set of CpG sites, the DNA methylation levels of which can be used to measure subject age - has unveiled novel molecular markers to monitor aging and assess a more accurate “epigenetic age”.

This Research Topic contributed to elucidating the role of DNA methylation and gene expression as a measure to understand and decipher aging in various disease contexts.

It is widely accepted that multiple environmental exposures and lifestyle factors, including obesity, can accelerate epigenetic aging, thus leading to an increased risk of age-related metabolic diseases (de Toro-Martín et al., 2019; Nannini et al., 2019; Fiorito et al., 2021; Etzel et al., 2022). Franzago et al. deeply review the interaction between epigenetic aging and obesity, emphasizing how the epigenome may serve as an intriguing target for age-related physiological changes and how its modification could influence aging and prolong a healthy lifespan. In particular, the authors focus on DNA methylation age as a clinical biomarker and on the potential reversal of epigenetic age using a personalized diet- and lifestyle-guided intervention (Franzago et al.).

Song et al. carry out an integrated analysis utilizing gene expression and DNA methylation microarrays in the temporal cortex of Alzheimer’s disease (AD) patients to further identify aberrantly methylated genes and define the molecular processes related to AD. Aging is a major risk factor for AD, the most prevalent cause of dementia, characterized by abnormal deposition of amyloid-β (Aβ) plaques and tau tangles in the brain associated with cognitive impairment. Several studies demonstrated that abnormally methylated genes have critical roles during AD neuropathology (Hass et al., 2009; Sanchez-Mut et al., 2014). Therein, the authors present numerous deregulated genes that are significantly enriched in biological processes such as cell morphogenesis, chemical synaptic transmission, and regulation of Aβ formation. In particular, they observe reduced methylation levels and increased *TGFBR3* expression associated with Aβ accumulation. The authors suggest TGFBR3 might promote Aβ production by enhancing *ß*- and *γ*-secretase activities and propose *TGFBR3* as a risk diagnostic biomarker and a therapeutic target for AD treatment (Song et al.).

Finally, Liu et al. investigate the impact of ethnicity on DNA methylation changes in peripheral blood cells from breast cancer (BC) patients. Age is one of the strongest risk factors for the development of BC, and evidence shows accelerated epigenetic aging in normal breast tissues adjacent to breast tumors in BC patients (Hofstatter et al., 2018; Rozenblit et al., 2022). DNA methylation signatures have been identified in the white blood cells as potential biomarkers for BC. In this writing, the authors identify relevant differences in the DNA methylation profiles when comparing European and Chinese populations. They report that BC is associated with altered methylation of *CD160*, *ISYNA1,* and *RAD51B* in the peripheral blood cells of European women, while an opposite profile is observed in the Chinese population. This may be due to genetic background or lifestyles and therefore warrant validation of epigenetic biomarkers in various ethnic groups (Liu et al.). However, large-scale prospective analyses will be needed to confirm the diagnostic and, or prognostic value of DNA methylation signatures in patients with BC, given the relatively small size of the analyzed case-control population.

In conclusion, by using different models and approaches, the studies presented in this Research Topic not only define DNA methylation as a tool to measure aging but identify this marker as a potential target to reverse age-related conditions, thereby reiterating the biological and clinical relevance of the DNA methylation to assess the aging process.
